# Supplementary material for: Determination of the Allelic Composition of the sdw1/denso (HvGA20ox2), uzu1 (HvBRI1) and ari-e (HvDep1) Genes in Spring Barley Accessions from the VIR Collection
Source: Plants (Basel). 2024 Jan 27;13(3):376. doi: 10.3390/plants13030376 (PMC10857314; doi:10.3390/plants13030376)
Supplement: Supplementary file 1 [file plants-13-00376-s001.zip › plants-2822515-supplementary.pdf]

Table S1. Primers used for identifying barley dwarfing alleles

Gene	Primer name	Marker type	Sequence 5'→3'	Target mutation	Target allele identification	Literature source
HvGA20ox2	Identification of *sdw1.c* and *sdw1.ins*					
	MC40861P3F	InDel	tatggcgtgaccaaaggttc	64 (+GTTA)	No PCR - *sdw1.a* or *sdw1.e*<br>247 bp - *sdw1.c*, 410 bp - *sdw1.ins*	[4]
	MC40861P4R		caccaatccaccacgaaga			
	Program: 94 °C – 3 min 30 sec; 37 cycles (94 °C – 45 sec, 54 °C – 45 sec, 72 °C – 1 min); 72 °C – 7 min					
	dCsdw1.c-F	dCAPS	cgtgaccaaaggttcctgtcc	64 (+GTTA)	No PCR - *sdw1.a* or *sdw1.e*<br>HpaII: CCGG[2]<br>276 bp – *sdw1.ins*<br>113 bp (site is absent) – *sdw1.c*	Present study
	dCsdw1.c-R		attgctgaggcggccatgtta**c**c[1]			
	Program: 94 °C – 3 min 30 sec; 37 cycles (94 °C – 45 sec, 61 °C – 45 sec, 72 °C – 30 sec); 72 °C – 7 min					
	Identification of *sdw1.d*					
	Hv20ox2-F	CAPS	cgctgattaactgggacaca	1665 G→A	No PCR - *sdw1.a* or *sdw1.e*<br>HaeIII: GGCC<br>194 bp (site is absent)	[6, 24]
	Hv20ox2-R		gttcgctcgttaggaagcag			
	Program: 94 °C – 3 min 30 sec; 8 cycles (94 °C – 45 sec, 59 °C with a 0.5 °C stepdown per 1 cycle – 1 min 30 sec, 72 °C – 1 min); 30 cycles (94 °C – 45 sec, 55 °C – 45 sec, 72 °C – 45 sec); 72 °C – 7 min					
HvBrI1	Identification of *uzu1.a*					
	–	dCAPS	gaaatggagaccattggcaagatcaa**g**c	2612 A→G	BstHHI: GCGC<br>265 + 29 bp (site is available)	[16]
	–		ccttgcctccagattctcatcaac			
	Program: 94 °C – 3 min 30 sec; 8 cycles (94 °C – 45 sec, 67 °C with a 0.5 °C stepdown per 1 cycle – 1 min 30 sec, 72 °C – 1 min); 30 cycles (94 °C – 45 sec, 63 °C – 45 sec, 72 °C – 45 sec); 72 °C – 7 min					
HvDep1	Identification of *ari-e.GP*					
	dCari-e.GP-F	dCAPS	agcagcaccaattctctcgtaa	1508 (+A)	FokI: CATCC<br>154 bp (site is absent)	Present study
	dCari-e.GP-R		caagcagaacgtgagactgg**a**t	*Fok*I: CATCC		
	Program: 94 °C – 3 min 30 sec; 37 cycles (94 °C – 45 sec, 60 °C – 45 sec, 72 °C – 30 sec); 72 °C – 7 min					

[1] Underlined are nucleotides changed in the primers (compared to the original gene sequence) for creating dCAPS markers.

[2] Instead of these restriction enzymes, their isoschisomers HpaII (BsiSI, HapII, MspI), HaeIII (BshFI, BsnI, BspANI, BsuRI), BstHHI (AspLEI, CfoI, Hin6I, HinP1I, HspAI), FokI (BseGI, BstF5I, BtsCI) can be used.

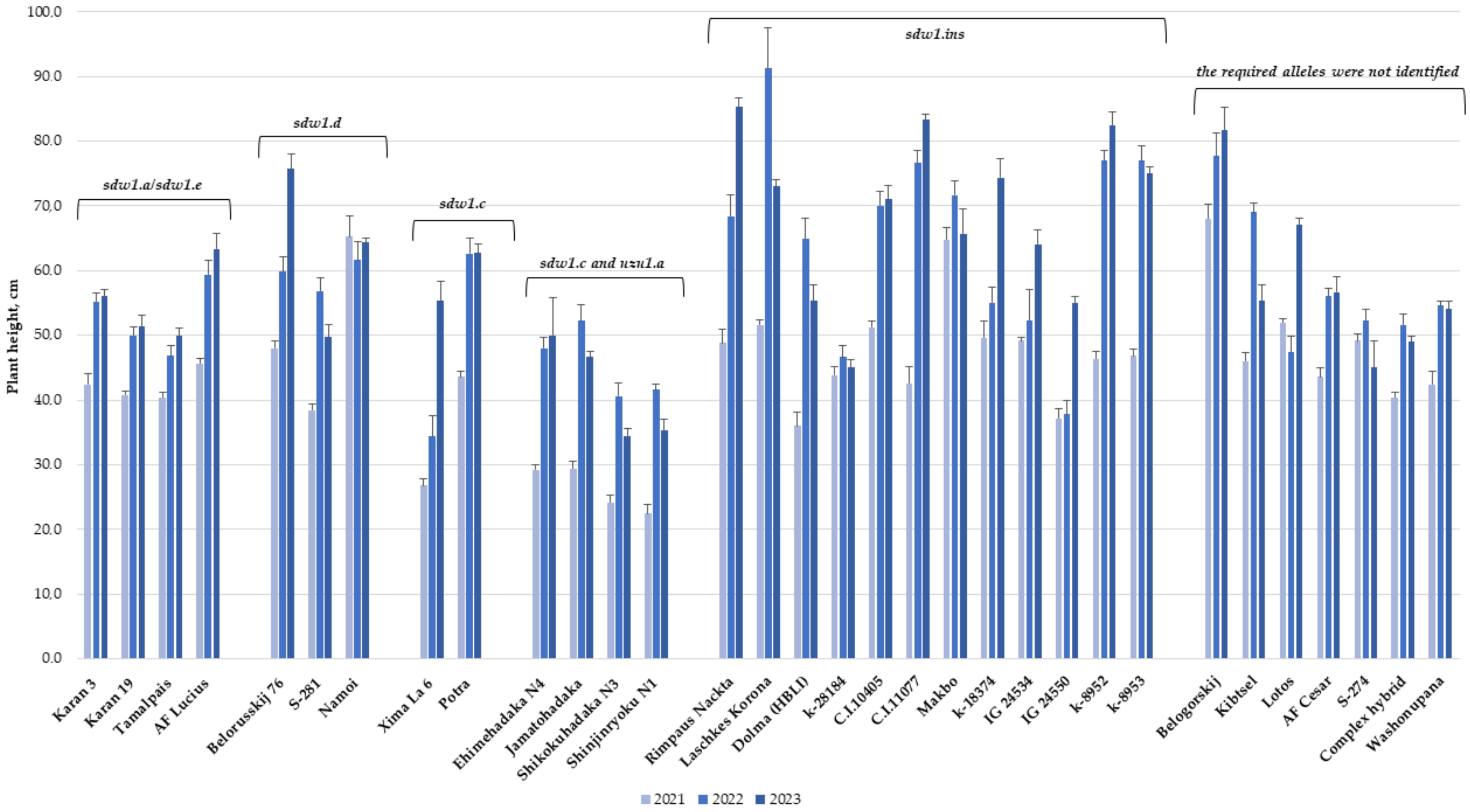

Figure S1. Variation in height of barley accessions in 2021-2023.

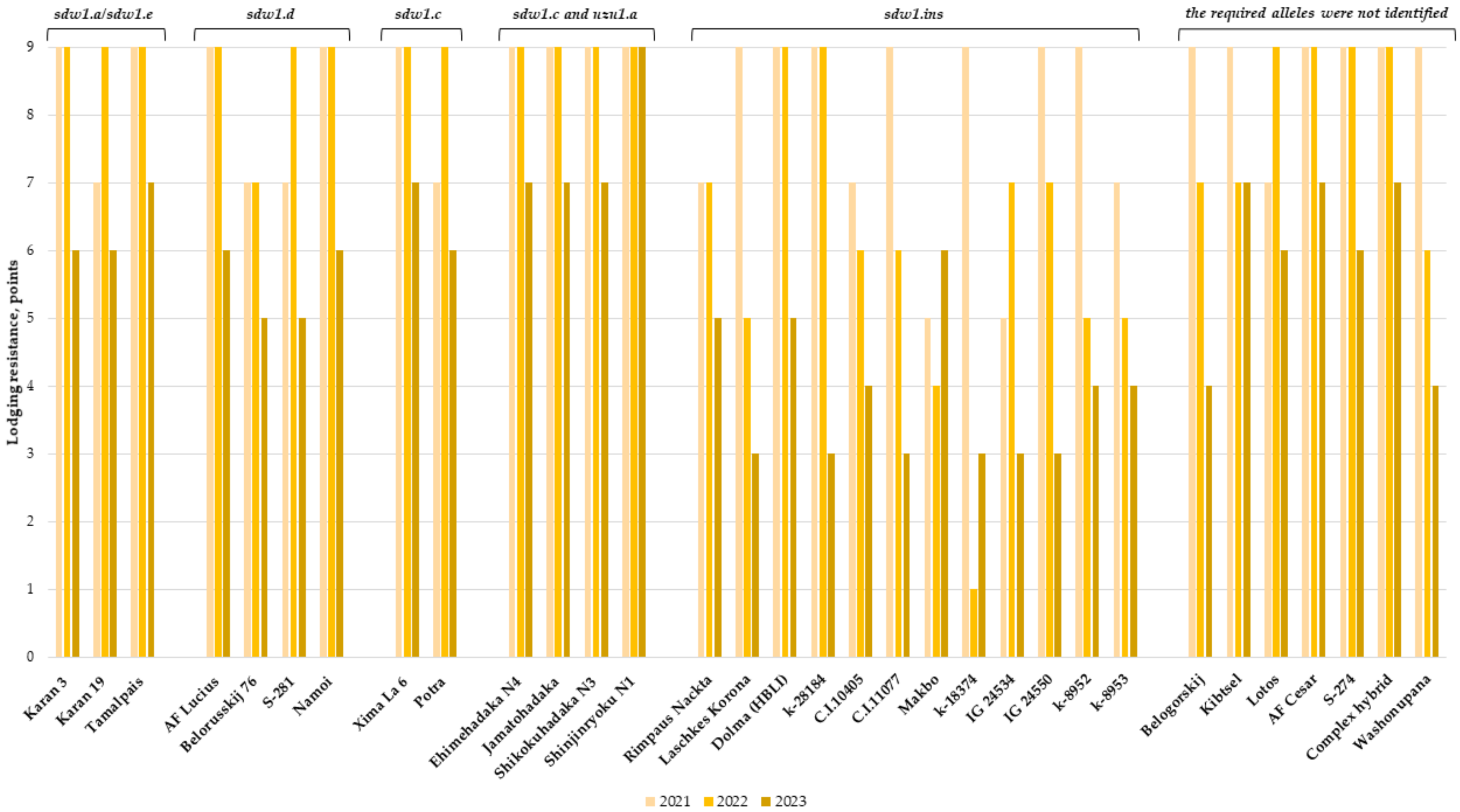

Figure S2. Variation in lodging resistance of barley accessions in 2021-2023 (9 points - high lodging resistance, 5 - average resistance, 1 – lodging).

```
                                            10        20        30        40        50        60
                                   ....|....|....|....|....|....|....|....|....|....|....|....|
                                   MC40861P3F ------->
wt (cv. Morex)                 1   TATGGCGTGACCAAAGGTTCCTGTCCGGTAAAGGAGGAGTCAGTATGCAGCTCAGTACCC 60
sdw1.c (cv. Deba Abed)         1   ............................................................ 60
sdw1.ins (VIR acc.)            1   ............................................................ 60
sdw1.ins (pan-genome acc.)     1   ............................................................ 60

                                            70        80        90       100       110       120
                                   ....|....|....|....|....|....|....|....|....|....|....|....|
wt(cv. Morex)                  61  GCAAAAATAAAAAATGCAGCTCACTGGTGTCCGTTA------------------------ 96
sdw1.c (cv. Deba Abed)         61  .............-......................------------------------ 95
sdw1.ins (VIR acc.)            61  ....................................CTCCCTCCGTAACTAAATATAATT 120
sdw1.ins (pan-genome acc.)     61  ....................................CTCCCTCCGTAACTAAATATAATT 120

                                           130       140       150       160       170       180
                                   ....|....|....|....|....|....|....|....|....|....|....|....|
wt (cv. Morex)                 96  ------------------------------------------------------------ 96
sdw1.c (cv. Deba Abed)         95  ------------------------------------------------------------ 95
sdw1.ins (VIR acc.)            121 CTTTTAAGATATTTCACTAGGTGTCTACATACGAAGCAAAGTGAGTGAATCTATACTTTA 180
sdw1.ins (pan-genome acc.)     121 CTTTTAAGATATTTCACTAGGTGTCTACATACGAAGCAAAGTGAGTGAATCTATACTTTA 180

                                           190       200       210       220       230       240
                                   ....|....|....|....|....|....|....|....|....|....|....|....|
wt (cv. Morex)                 96  ------------------------------------------------------------ 96
sdw1.c (cv. Deba Abed)         95  ------------------------------------------------------------ 95
sdw1.ins (VIR acc.)            181 AATTATGTCTATATACATCCGTATGTAGTTCATTAGTGAAACCTGTAGAAAGACTTATAT 240
sdw1.ins (pan-genome acc.)     181 AATTATGTCTATATACATCCGTATGTAGTTCATTAGTGAAACCTGTAGAAAGACTTATAT 240

                                           250       260       270       280       290       300
                                   ....|....|....|....|....|....|....|....|....|....|....|....|
wt (cv. Morex)                 96  ----------------------ACATGGCCGCCTCAGCAATATCCTAATCACCATCCATT 134
sdw1.c (cv. Deba Abed)         95  ------------------GTTA...................................... 137
sdw1.ins (VIR acc.)            241 TTAAGAACGGAGGGAGTAGTTA...................................... 300
sdw1.ins (pan-genome acc.)     241 TTAAGAACGGAGGGAGTAGTTA...................................... 300

                                           310       320       330       340       350       360
                                   ....|....|....|....|....|....|....|....|....|....|....|....|
wt (cv. Morex)                 135 TGTGACCTTATTACGAGTCTCAAAGTAGGATCGGACGGTGATGGAGGTAGCCCCTGTAGG 194
sdw1.c (cv. Deba Abed)         138 ............................................................ 197
sdw1.ins (VIR acc.)            301 .................................A.......................... 360
sdw1.ins (pan-genome acc.)     301 .................................A.......................... 360

                                           370       380       390       400       410
                                   ....|....|....|....|....|....|....|....|....|....|
                                                                <------- MC40861P3R
wt (cv. Morex)                 195 GATCAAACCCGGAAAAGCTTTTATCCCATTCTCTTCGTGGTGGATTGGTG 244
sdw1.c (cv. Deba Abed)         198 .................................................. 247
sdw1.ins (VIR acc.)            361 .................................................. 410
sdw1.ins (pan-genome acc.)     361 .................................................. 410
```

Figure S3. Sequence alignment of PCR-products flanked by the MC40861P3F/R primers. The sequences homologous to the Thalos_2 transposon are highlighted in bold. VIR accessions are Dolma, Makbo, Rimpaus Nackta, C.I.10405 and k-8953; pan-genome accessions are HOR8148, HOR10350 and HOR13821 with the same sequences.
